# Targeting Chemokines and Chemokine Receptors in Melanoma and Other Cancers

**DOI:** 10.3389/fimmu.2018.02480

**Published:** 2018-10-29

**Authors:** Nicolas Jacquelot, Connie P. M. Duong, Gabrielle T. Belz, Laurence Zitvogel

**Affiliations:** ^1^Walter and Eliza Hall Institute of Medical Research, Melbourne, VIC, Australia; ^2^Department of Medical Biology, University of Melbourne, Melbourne, VIC, Australia; ^3^Gustave Roussy Comprehensive Cancer Institute, Villejuif, France; ^4^INSERM U1015, Villejuif, France; ^5^Faculty of Medicine, Paris Sud/Paris XI University, LeKremlin-Bicêtre, France; ^6^Center of Clinical Investigations in Biotherapies of Cancer (CICBT) 1428, Villejuif, France

**Keywords:** chemokine, chemokine receptor, melanoma, immune cell trafficking, cell migration

## Abstract

The tumor microenvironment is highly heterogeneous. It is composed of a diverse array of immune cells that are recruited continuously into lesions. They are guided into the tumor through interactions between chemokines and their receptors. A variety of chemokine receptors are expressed on the surface of both tumor and immune cells rendering them sensitive to multiple stimuli that can subsequently influence their migration and function. These features significantly impact tumor fate and are critical in melanoma control and progression. Indeed, particular chemokine receptors expressed on tumor and immune cells are strongly associated with patient prognosis. Thus, potential targeting of chemokine receptors is highly attractive as a means to quench or eliminate unconstrained tumor cell growth.

## Introduction

**Figure 1:**
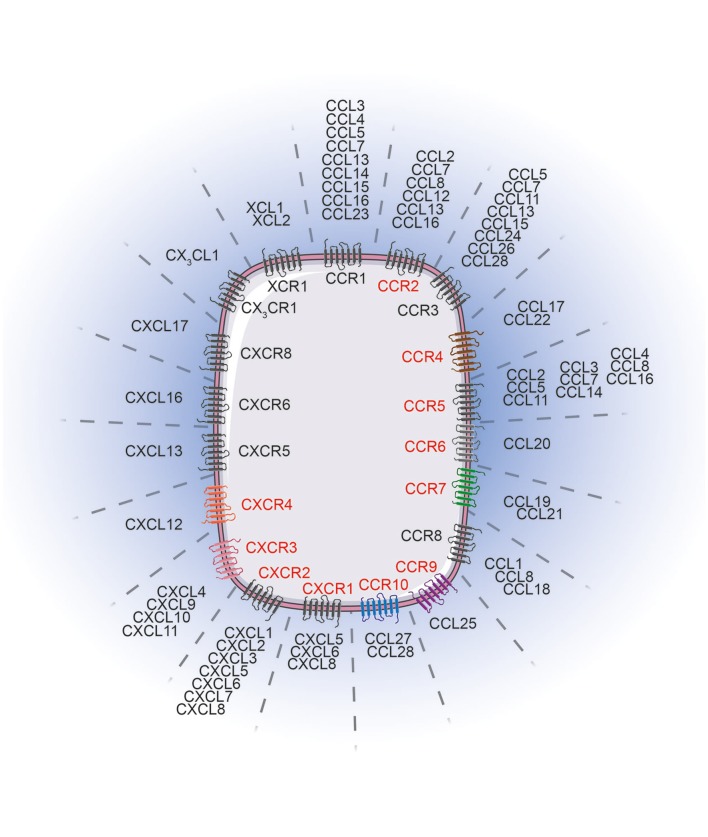
Chemokine receptors and their corresponding ligands. Chemokine receptors (red) influence melanoma tumor cell migration/invasion or immune cell trafficking to the tumor lesions. The chemokine receptor associated color code is conserved between Figures 1, 2. Images were taken from Servier Medical Art (https://smart.servier.com) and modified by the authors under the following terms: Creative Commons Attribution 3.0 Unported License.

Patient outcome is dictated by the capacity of immune cells to mount an effective anti-tumor response. Migration to, and infiltration of, tumors by immune cells is critical for achieving this goal. Elevated tumor immune infiltration is often associated with a favorable prognosis in many malignancies (1–3) including melanoma (4–6). Although fundamental in the anti-tumor immune response, tumor infiltration by immune cells is a challenging process. Immune cells are guided from the circulation to the tumor microenvironment by an evolutionarily conserved and sophisticated system in the form of the chemokine network. Chemokines are cytokines with chemotactic properties. This superfamily consists of 48 proteins classified into 4 groups (XCL, CCL, CXCL, and CX3CL) based on the position of two cysteine residues in their sequence. They bind to 19 G protein-coupled seven transmembrane receptors that form either homodimers or heterodimers (7–11). Similar to their ligands, chemokine receptors are classified into 4 groups, namely XCR, CCR, CXCR, and CX3CR. Each receptor can bind to several ligands of the same family and vice versa (Figure 1). Beyond this, atypical chemokine receptors also exist and most act as decoy receptors that compete for ligand binding but are unable to deliver normal chemokine receptor signals. They serve as negative regulators during inflammatory responses (12). The expression of these receptors and ligands is finely regulated, both spatially and temporally, revealing distinct functions at steady-state and during inflammatory responses. Many chemokines are constantly expressed and participate in the maintenance of tissue integrity, while some chemokines are transiently overexpressed or specifically induced in certain conditions (i.e., during inflammatory processes) where they are involved in critical biological functions (i.e., immune cell migration, tissue repair, cell proliferation and angiogenesis) (10, 13, 14). Both immune and non-immune cells express these receptors and ligands, and the impact of this expression differs according to cell types. On one hand, selective expression drives the recruitment of specific immune cells into tumors, subsequently influencing patient prognosis. On the other hand, overexpression of chemokine receptors on cancer cells facilitates tumor dissemination. Collectively, dysregulation of this tightly regulated system contributes to tumor escape, and therefore, appears to be an attractive target in melanoma and other cancers.

Here, we review the expression of chemokines and chemokine receptors critically involved in skin migration, their expression on immune and tumor cells and consequences on dictating patient prognosis and, finally, their potential of targeting in melanoma and other cancers.

## Migration to the skin

The skin forms a physical barrier between an organism and the environment. It is mainly composed of melanin-producing cells, melanocytes, epithelial cells, keratinocytes, stromal cells, and immune cells that play critical roles both in maintaining homeostasis with commensals and in rapidly detecting and limiting pathogen infection and dissemination. Several immune cell types reside in the skin and act as essential sentinels (15). These include memory T cells, Langerhans cells and other types of dendritic cells (DC), macrophages, mast cells and innate lymphoid cells that collectively form a dense network that underlies the entire skin surface (15, 16). Localized at the frontline, keratinocytes are fundamental in protecting us against infections. They express different receptors, called pattern recognition receptors, specialized in the identification of conserved motifs across microorganisms (17). Upon detection of an infection or even after injury, activated keratinocytes start to secrete antimicrobial peptides, pro-inflammatory cytokines and chemokines (14, 15, 18, 19). In response to this local accumulation of chemokines and particularly to CXCL8, CXCL1, CCL2, CCL3 and CCL5, CXCR2-expressing monocytes and neutrophils are attracted to the inflammatory site and amplify this initial response (10, 15). Moreover, neutrophils are also attracted to the skin via binding of surface expressed formyl peptide receptor 1, to formylated peptides released by pathogens or dead or dying cells (20). In parallel, skin-resident DC drive immune responses through their potential to take up antigens. This process induces DC maturation and activation leading to membrane expression of CCR7 and CXCR4. In addition, this expression provokes their migration from the skin to the closest skin-draining lymph node (10, 21). Antigen-specific T cells are imprinted with skin-homing molecules following their engagement with, and activation by, primed DC. These homing molecules include CCR3, CCR4, CCR5, CCR10, CXCR3, and Cutaneous Lymphocyte associated Antigen (CLA), a ligand for E-selectin (22–25). The expression of these receptors facilitates T cell migration to the skin through binding of E-selectin that is expressed selectively on activated skin endothelial cells (22, 26). Moreover, together with skin-resident cells, these endothelial cells also secrete specific chemokines such as CCL17, CCL20, CCL22 and CCL27, ligand of CCR4, CCR6, and CCR10, respectively, that guide these antigen-specific T cells specifically to the inflamed skin lesion (15, 27–31). This migratory pathway is essential for wound healing after skin injury and for efficient elimination of infections. In addition, these chemokine—chemokine receptor interactions are also of extreme importance in melanoma immunity. Primary tumors localized in the skin are continuously evolving as a result of the constant infiltration to, and egress of cells from, the microenvironment. This is facilitated by the presence of blood and lymphatic vessels that guide immune cells to the tumor bed but also enable cancer cells to disseminate to various organs. Chemokines and their receptors are critically involved in these migratory processes and actively control the specific metastatic melanoma landscape.

## Specific chemokine receptor expression on melanoma cells is associated with distinct metastatic dissemination

**Figure 2:**
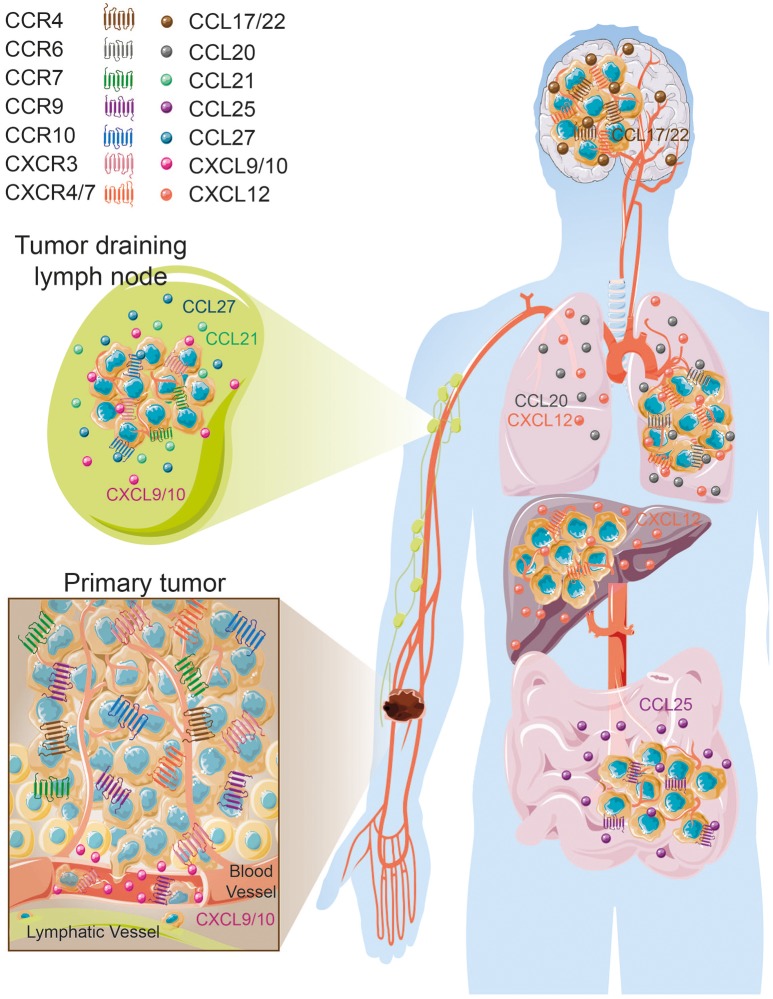
Organ-specific melanoma metastases according to tissue/melanoma specific chemokine/chemokine receptor expression. Images were taken from Servier Medical Art (https://smart.servier.com) and modified by the authors under the following terms: Creative Commons Attribution 3.0 Unported License.

The formation of secondary lesions involves two major steps. First, tumor cells are guided from the circulation to their final location in response to a chemokine gradient expressed in different organs and then, these newly seeded tumor cells must survive and proliferate in these specific environments subsequently forming distant metastases (9, 32). In cutaneous melanoma, as a result of a specific chemokine receptor expression pattern, melanoma cells disseminate in an organ-specific manner that forms secondary lesions preferentially in draining lymph nodes, lung, liver, gut and brain (Figure 2) (33, 34). To determine the role of key chemokine receptors in tumor cell migration in melanoma, many of the mouse studies described here have used the prototypic mouse melanoma model, B16, or its highly metastatic subclone B16F10 (35, 36). The combination of preclinical studies and retrospective assessment of human melanoma samples for chemokine receptor expression have shed light on a finely controlled process that notably involves CCR4, CCR6, CCR7, CCR9, CCR10, CXCR3, CXCR4, and CXCR7 expression.

### CCR4–CCL17/CCL22 axis

Several lines of evidence evoked by Klein et al. (37) tend to associate CCR4 expression with increased brain melanoma metastases (37). Endothelial cells, astrocytes and microglia cells were shown to express high levels of CCR4 ligands, CCL17 and CCL22 (37) that likely attract CCR4^+^ cells. *In vitro* incubation of microglia cells with conditioned media from brain metastasizing melanoma cells increased CCR4 ligand secretion. Furthermore, CCR4 is more highly expressed on melanoma brain metastases than on paired-primary melanoma tumors (37) (Figure 2). Klein et al. (37) have further studied whether CCR4 overexpression in melanoma cells favor brain metastasis formation. *In vitro*, CCR4 overexpression enhanced cell viability and migration in response to astrocyte-conditioned media and to recombinant CCL17. This migration is partially abrogated by the concomitant use of an anti-CCL17 antibody. *In vivo*, CCR4 overexpression promoted primary tumor growth and enhanced brain metastases formation in immunocompromised nude mice. Importantly, mice inoculated with CCR4^high^ expressing tumor cells and treated with a CCR4 antagonist had a significant reduction of primary tumor growth associated with a decrease of the presence of brain micrometastases (37). Collectively these results suggest that CCR4 overexpression on melanoma tumors might enhance their potential to metastasize to the brain (Table 1, Figure 2).

**Table 1:** Expression of chemokine receptors at the surface of melanoma cells involved in tumor progression.

**Chemokine Receptor**	**Roles in tumor development/progression**	**Clinical association**	**Cohort details**	**Statistical analyses**	**References**
CCR4	Favor tumor cell viability, migration, primary tumor growth, and brain metastases formation	Not known	*In vitro* and preclinical models		(37)
CCR6	Enhanced tumor cell migration, proliferation, tumor growth, and lung metastasis formation	Not associated with patient outcome^*^	40 primary melanomas	Log-rank and Cox regression	(38)
CCR7	Associated with regional lymph node metastases	Poor prognosis	Preclinical model and 38 primary human samples	Log rank test—*P* = 0.009	(39, 40)
CCR9	Expressed on tumor cells localized in the small intestine–Sensitive to CCL25 stimulation	Not associated with patient outcome^*^ or not assessed	38 primary samples	Log rank test	(40–42)
CCR10	Associated with an increase of regional lymph node metastases, metastatic sentinel lymph node, thickening of primary lesions and poor T cell density	Shorter progression free survival	40 primary lesions and 38 primary melanoma samples	Spearman correlation and Log rank test–*P* = 0.002	(40, 43, 44)
CXCR3	Associated with thick primary lesions, the absence of lymphocytic infiltration and the presence of distant metastases—Increase in cell adhesion, migration, and invasion of CXCR3 expressing melanoma cells lines upon stimulation.	Not associated with patient outcome^*^	Primary melanomas and 9 Lymph node metastases	χ^2^, Mann-Whitney U and Kruskal Wallis tests—Log-rank test and Cox regression	(45–48)
CXCR4	Associated with the presence of ulceration, thicker lesions—Induce tumor cell proliferation, migration, and invasion—Associated with liver and lung metastases	Reduced disease-free and overall survival	Primary melanomas and metastatic samples	χ^2^ 2-sided test—Log-rank test and Cox regression	(47, 49–52)

* *Complementary analyses on larger cohorts are warranted*.

### CCR6–CCL20 axis

CCR6 is expressed on melanoma cell lines and enhances their migration and proliferation in response to stimulation by its ligand, CCL20 (38). Importantly, CCR6 expression is detected on tumor cells from primary melanomas, lymph node, skin, colon, and brain metastases. Despite high expression on tumor cells, CCR6 positivity is not associated with patient outcome. However, CCL20 administration in CCR6^+^ tumor bearing mice increased tumor weight and numbers of spontaneous lung metastases (38) (Table 1, Figure 2) suggesting the potential involvement of CCR6 in lung metastasis formation. Interestingly, Fusi et al. (53) have evaluated the presence of CCR6 expression on circulating tumor cells collected from metastatic carcinoma (*N* = 28) and melanoma (*N* = 21) patients. Positive CCR6 expression on circulating tumor cells, evaluated on the whole cohort, was not found to be associated with the presence of lung metastases (53). However, this chemokine receptor might be regulated differently according to tumor type. Thus, further studies are required to understand the impact of tumoral CCR6 expression in metastatic dissemination and how this chemokine receptor might influence melanoma outcome.

### CCR7–CCL19/CCL21 axis

Kuhnelt-Leddihn et al. have shown that 6 out of 38 primary melanoma tumors evaluated presented with high CCR7 expression (40), a chemokine receptor involved in leukocyte trafficking to secondary lymphoid organs in response to the local production of CCL19 and CCL21 (Table 1, Figure 2). CCR7 has also been found on circulating tumor cells and human metastatic melanoma cell lines (51, 53). Treatment of metastatic melanoma-derived cell lines with histone deacetylase inhibitor and demethylating agents demonstrated that this increase in CCR7 expression is associated with the enhanced migratory responses to CCL21 stimulation (54). Interestingly, CCL21 expression is decreased in invaded lymph node compared to non-invaded lymph node (55) that may suggest an escape mechanism to avoid tumor immune infiltration, specifically by CCR7 expressing T cells and DC (10, 56). In mice, overexpression of CCR7 in B16 melanoma cells increased metastasis to the lymph node and neutralizing its ligand, CCL21, using a specific antibody blocked this metastatic process (39), highlighting the importance of this CCR7/CCL21 axis in the metastasis to the regional lymph node. Overexpression of CCL21 in tumor cells induce a tolerogenic microenvironment associated with a production of Transforming Growth Factor-β (TGF-β) that favors the recruitment of regulatory T cells (Tregs) and myeloid deriving suppressor cells (MDSC) (57). More importantly, high expression of CCR7 by melanoma cells is associated with a worse patient outcome (40) (Table 1).

### CCR9–CCL25 axis

CCR9 is a chemokine receptor involved in the migration of T cells and other immune cells to its ligand, CCL25, which is highly expressed in the small intestine (58). Melanoma tumor cells that have metastasized to the small intestine have been shown to express CCR9 (41, 42) (Table 1, Figure 2). Importantly, CCR9^+^ melanoma cell lines derived from small intestinal metastases are responsive to CCL25 (41, 42). CCR9 expression has been also reported on circulating tumor cells (53). Unfortunately, the association between CCR9 expression on circulating tumor cells and small intestine metastases has not been assessed. Moreover, after screening a panel of 38 primary melanoma tumors, CCR9 expression was not found to be associated with patient's prognosis despite being highly expressed in one third of lesions (40). Collectively, these results suggest that CCR9 expression at the surface of melanoma cells may be essential for the migratory process to the gut (Figure 2).

### CCR10–CCL27 axis

CCR10 is expressed on melanoma cells in primary tumor lesions (40, 43). Using a preclinical model of melanoma, overexpression of CCR10 in B16 tumor cells protected them from the host immune responses leading to an increase in tumor size and increased regional lymph node metastases (43). Incubating tumor cells with a neutralizing antibody for CCL27, one of the ligands of CCR10, prevented tumor formation (43). These results indicate that CCR10 may play an important role in sustaining tumor viability, protecting cells from immune responses and favoring metastases formation to the regional draining lymph node in response to CCL27. In humans, high CCR10 expression may be associated with a shorter progression free survival (40) (Table 1). Strikingly, patients with metastatic sentinel lymph nodes had higher levels of CCR10 expression on primary tumor cells than patients with negative sentinel lymph node (44). This observation further supports the probable role of this chemokine receptor in regional lymph node dissemination (Figure 2). Moreover, high CCR10 expression was associated with thick primary lesions and negatively correlated with intratumoral T cell density (44) (Table 1). Altogether, CCR10 overexpression on melanoma cells is associated with the possible presence of regional lymph node metastases (Figure 2) accompanied by an immune negative climate.

### CXCR3–CXCL9/CXCL10 axis

CXCR3 expression on primary lesion tumor cells is positively associated with deleterious clinical parameters including thickening of primary lesions, absence of lymphocytic infiltration, and presence of distant metastases (47, 48) but, surprisingly, is not correlated with patient outcomes (48). Nonetheless, high CXCR3 expression evaluated on 40 primary melanoma tumors tended to be associated with poor disease-free and overall survivals (48). CXCR3 positive tumor cells are also found in invaded lymph nodes (Figure 2) and together with other metastatic locations including the kidney, ovary and pleura (45, 59). Interestingly, tumor endothelial cells facilitate melanoma migration through their production of CXCL9 (and CXCL10). This results in endothelial barrier disruption and transendothelial migration (59) (Figure 2). In addition, *in vitro* stimulation of melanoma cell lines with CXCL9 induced cytoskeletal rearrangements, cell adhesion and migration (45), that favor cell trafficking and metastasis. Similarly, *in vitro* incubation of the mouse melanoma cell line B16F10 with CXCR3 ligands significantly enhanced migration and invasion of these cells (46). Conversely, specific downregulation of CXCR3 in subcutaneous injected B16F10 tumor cells reduced their metastatic capabilities to invade the tumor draining lymph node (46). Mouse melanoma tumor cells incubated with the CXCR3 ligand, CXCL9, exhibited greater viability than the control cells (Table 1), thus demonstrating that CXCR3 imparts a selective advantage to tumor cells most likely allowing them to compete more effectively for oxygen and nutrient availability in the competitive tumor microenvironment (60–62).

### CXCR4/CXCR7–CXCL12 axis

In primary skin tumors, cancer cells express CXCR4, a chemokine receptor involved in bone marrow homing and cell retention (10). Importantly, high CXCR4 expression is associated with the presence of tumor ulceration and thicker lesions, as well as shorter disease-free survival, time to metastasis and overall survival (47, 63) (Table 1). Tumoral CXCR4 expression has also been detected on circulating tumor cells (53) as well as in liver, lung, and nodal metastases (49, 51). Using melanoma cell lines, Scala et al. demonstrated that these cells express functional CXCR4, as *in vitro* stimulation with CXCL12 in serum free media increased their proliferation that was abrogated with the concomitant use of a CXCR4 inhibitor, AMD3100 (51). The B16 mouse melanoma cell line constitutively expresses CXCR4. This increased the cell migration, invasion and proliferation in response to the binding its ligand, CXCL12 (52). Importantly, CXCL12 stimulation induced cell adhesion to liver sinusoidal endothelial cells and *in vivo*, B16 liver metastases are often localized to CXCL12 expressing liver sinusoidal endothelial cells. Mendt and Cardier (52) have shown that stimulation of B16 cells with CXCL12 prior *in vivo* injection increased the number of liver metastases (52). Several lines of evidence tend to also involve the CXCR4-CXCL12 pathway in lung metastasis formation. Firstly, high CXCL12 concentrations are found in lungs (64). Secondly, overexpression of CXCR4 in B16 cells enhanced lung nodules formation (49, 50, 65) (Table 1). Thirdly, the use of specific CXCR4 inhibitors, T22 or a dimeric form of CXCL12, reduced lung metastases formation and inhibited the growth of primary melanoma tumors (49, 66, 67). However, CXCR4 expression on circulating tumor cells was not found preferentially associated with liver metastases or with lung metastases in metastatic carcinoma or melanoma patients (53).

CXCL12 also binds to its high-affinity receptor CXCR7, an atypical chemokine receptor also known as ACKR3. CXCR7 is expressed on normal human epidermal melanocytes (68) and primary melanoma tumors (63, 69). The role and functions of CXCR7 in cell migration/chemotaxis is still controversial (70). In neuroblastoma cell lines, overexpression of CXCR7 was shown to limit cell growth and CXCR4/CXCL12-mediated chemotaxis (71). In contrast, some studies have demonstrated that CXCR7 expression favors hepatocellular carcinoma cell proliferation, migration and VEGF production (72), transendothelial migration of cancer cells (73, 74), and tumor cell migration by forming heterodimers with CXCR4 (75). Using the M14 melanoma cell line that expresses functional CXCR7, Li et al. have demonstrated that *in vitro* incubation of M14 cells with CXCL12 induced cell migration, which was specifically reduced following abrogation of CXCR7 expression (69). Furthermore, downregulation of CXCR7 expression in the melanoma cell line decreased the growth of the xenotransplanted tumor. However, the expression of CXCR4 was not reported in this study. The full deletion of CXCR4 in M14 cells together with the modulation of CXCR7 expression are warranted in order to definitively determine the impact of this atypical chemokine receptor on M14 cell growth and migration. Furthermore, its expression on melanoma metastases and its association with patient prognosis remain to be determined. Altogether, CXCR4 is involved in the metastatic spreading of melanoma cells and therefore may influence patient outcomes. Based on pre-clinical results, it is also tempting to say that tumoral CXCR4 expression is more preferentially associated with lung and liver metastases (Table 1, Figure 2). However, additional studies are warranted to determine the involvement of the CXCR4/CXCR7 -CXCL12 axis in favoring organ-specific metastasis formation as reported in breast or colorectal cancer (76–79).

In the past 20 years, numerous studies have demonstrated the pivotal role of these chemokine receptors in melanoma dissemination and how this coordinated chemokine receptor expression on the surface of melanoma cells is preferentially associated with specific organ metastases (9, 50, 80). CCR10, CCR7, and CXCR3 are found mainly involved in regional metastases formation while CCR9 is often associated with the intestine, CCR6 or CXCR4 are preferentially implicated in the formation of lung and liver lesions. CCR4 does however seem to be associated with brain metastases, which considerably impacts patient prognosis (81) (Table 1, Figure 2). Collectively, tumor cells eventually use these chemokines and chemokine receptors to their own advantage to be guided through the body to invade distant organs and create secondary lesions.

## Chemokine receptor expression on immune cells – decisive roles in melanoma lesion infiltration and tumor fate

Tumor immune cell infiltration is critical in dictating melanoma patient outcome (82–84). Specific expression of chemokine/chemokine receptors and integrins is fundamental to this process and is involved in the guidance and tissue retention of immune cells. Transcriptomic analyses of 569 cutaneous samples and 120 melanoma metastases have demonstrated the positive association of 12 chemokines (CCL2, CCL3, CCL4, CCL5, CCL8, CCL18, CCL19, CCL21, CXCL9, CXCL10, CXCL11, and CXCL13) with the presence of tertiary lymphoid structures, ectopic lymph node-like structures containing antigen presenting cells, B cells and T cells (85). This chemokine signature was associated with a favorable prognosis irrespective of tumor localization. This has been further validated in patients harboring primary tumors that contain peritumoral matured DC in combination with activated T lymphocytes (86). Furthermore, Harlin et al. found that a restricted signature of six chemokines, CCL2, CCL3, CCL4, CCL5, CXCL9, and CXCL10, were preferentially expressed in melanoma metastases that were highly infiltrated by T cells (87). Importantly, high gene expression of *Cxcl2, Cxcl9, Cxcl10*, and *Ccl5* together with *Ifn*γ, *Stat1*, and *Irf1* expression have been associated with the efficacy of MAGE-A3 vaccination (88) and with clinical responses to CTLA-4 blockade (89). Collectively, chemokines profoundly affect tumor immune cell composition and melanoma responses irrespective of tumor location. To date, the evaluation of these chemokines are not yet considered in daily clinical practice but they are likely to be essential to more accurately evaluate the prognosis of melanoma patients and/or therapeutic responses. Immune cell trafficking occurs after specific interactions between chemokines with their receptors that guide the immune cells to their final location. Thus, this expression is extremely important and dictates the tumor microenvironment diversity, considerably influencing melanoma evolution.

### CCR4–CCL2 axis

In human and mouse melanomas, the presence of Foxp3^+^ cells, mainly Tregs, in primary and metastatic tumors was associated with a poor prognosis (83, 90–93). Effector and regulatory T cells both express CCR4 but Foxp3^+^ Tregs expressed higher levels of CCR4 than their Foxp3^−^ effector T cell counterparts. Salerno et al. (94) have described the accumulation of CCR4^+^ effector CD4^+^ T cells, but not CD8^+^ T cells, in skin and bowel melanoma metastases (94). Given the large proportion of Tregs within the CD4^+^ population in tumor lesions, it is tempting to associate the presence of CCR4^+^ effector cells to Tregs. These cells migrated to the tumor bed in response to CCL2 accumulation (95–97). The use of an anti-CCR4 antibody *in vitro* efficiently reduced Tregs numbers enabling the induction of cancer/testis antigen-specific T cell responses (97) (Table 2). In pre-clinical models, the use of an anti-CD25 antibody, or Foxp3DTR (Diphtheria Toxin Receptor) mice where Foxp3-expressing cells can be inducibly deleted following diphtheria toxin injection, delayed tumor growth (100). However, in transgenic mouse melanoma models, the removal of Tregs was not sufficient to induce clinical improvements (96) suggesting that other immunosuppressive pathways are acting in concert to suppress anti-tumor immune functions. Moreover, in a therapeutic setting, anti-CD25 antibody injection did not reduce Treg proportions in tumors (96) potentially explaining the absence of clinical activity from the treatment.

**Table 2:** Expression of chemokine and chemokine receptors by immune cells associated with melanoma control or progression.

**Chemokine receptor**	**Immune cell expression**	**Roles in melanoma development/progression**	**Cohort details**	**Statistical analyses**	**References**
CCR2	Tumor macrophages and MDSC	Neutralization decreased tumor macrophage accumulations associated with a reduction of tumor angiogenesis and tumor growth	Preclinical studies		(98, 99)
CCR4	Blood and tumor Tregs	Depletion enhanced anti tumor immune responses. Controversial using the spontaneous *Ret* melanoma model.	*In vitro* and preclinical studies		(96, 97, 100)
CCR5	Blood and tumor Tregs and MDSC	CCR5^Δ32^ polymorphism in patients receiving immunotherapy associated with decreased survival Immunosuppression -Neutralization resulted in increased survival of tumor bearing mice	139 stage IV patients Preclinical studies	Log-rank test and Cox regression– *P* = 0.002	(101–104)
CCR6	Blood and tumor pDC—Blood CD8^+^ T cells	Higher expression in melanoma patients—circulating effector CCR6^+^CD8^+^ T cells and CCL20 expressed by tumor-associated macrophages conveyed a dismal prognosis	40 primary melanomas−57 stage III-IV patients	Log rank test and Cox regression	(38, 105, 106)
CCR9	Blood CD8^+^ T_Naive_	Associated with increased overall survival	57 stage III-IV patients	Log-rank test and Cox regression–*P* = 0.0036 (Stage-adjusted)	(106)
CCR10	Blood CD4^+^ T_EM_	Associated with worse survival	57 stage III-IV patients	Log-rank test and Cox regression–*P* = 0.0189 (Stage-adjusted)	(106)
CXCR2	Tumor MDSC and neutrophils	Accumulation of tumor CXCR2^+^ MDSC and neutrophils. CXCR2 neutralization reduced tumor growth	Preclinical studies		(107, 108)
CXCR3	Blood and tumor CD4^+^ and CD8^+^ T_EM_	Critical in intratumoral T cell trafficking—Associated with clinical benefit	Preclinical studies–Stage III-IV patients	Log-rank test, χ^2^ and Cox regression	(87, 106, 109, 110)
CXCR4	Blood CD45RA^+^CD4^+^ T cells	Associated with prolonged disease free survival	195 stage I-III patients	Log-rank test and Cox regression–*P* = 0.0091	(111)

*T_EM_: Effector memory T cells*.

### CCR5–CCL3/CCL4/CCL5 axis

The relationship between CCR5 expression on immune cells and tumor fate is not clear. In humans, little is known about the impact of CCR5 expression on immune cells and its association with patient outcomes. High CCR5 expression has been found on the surface of tumor infiltrating T cells (94). Interestingly, stage IV melanoma patients carrying a 32-bp –deletion polymorphism in the *Ccr5* gene, rendering this protein non-functional, have decreased survival following interferon treatment, interleukin-2 administration, or vaccination (101) suggesting a potential benefit of CCR5 expression in these specific settings. However, the use of CCR5-deficient mice, blockade antibody or CCR5-Ig fusion protein that acts as a decoy receptor neutralizing the CCR5 ligands, led to delayed tumor growth and increased the survival of these animals compared with control groups (102–104). Thus, CCR5 expression appears to be deleterious in pre-clinical models. CCR5 is highly expressed on tumor infiltrating CD8^+^ T cells, conventional and regulatory CD4^+^ T cells (102), and on the surface of MDSC (104). Importantly, CCR5^+^ MDSC displayed a more suppressive phenotype than their CCR5^−^ counterparts, expressing higher levels of Arginase 1 and producing more reactive oxygen species. The CCR5 ligands, CCL3, CCL4, and CCL5, are produced by intratumoral and circulating MDSC (102), acting in an autocrine manner on CCR5^+^ cells. Clinical improvements observed in CCR5-deficient mice or using CCR5 blockade were associated with a reduction of Tregs (102) and MDSC infiltration (103) together with a decrease of their immunosuppressive activities (104). In these models, conventional CD4^+^ and CD8^+^ T cell infiltration were maintained suggesting that CCR5 expression on the surface of these cells is not required for tumor infiltration (102). This observation has been confirmed by Mikucki et al. (110). Indeed, they demonstrated that the presence of CCR5 on CD8^+^ T cells was not essential for tumor infiltration despite high CCR5 ligand levels found in the tumor microenvironment (110). However, it remains unclear why MDSCs needs CCR5 expression for tumor infiltration, whereas T cells do not. In humans, both circulating monocytic (CD14^+^) and polymorphonuclear MDSC (CD15^+^CD11b^+^HLA-DR^lo/−^) express higher amounts of CCR5 on their membrane, compared to levels observed in healthy volunteers (104). Interestingly, CCR5 is more highly expressed on tumor infiltrating monocytic MDSC than on peripheral cells and high concentrations of CCL3, CCL4, and CCL5 are found in melanoma lesions, potentially explaining the enrichment of CCR5^+^ MDSC in tumors (104). Collectively, CCR5 expression sustains MDSC suppression activities, intratumoral Treg infiltration, and melanoma tumor growth (Table 2). Further studies in patients are needed to investigate the impact of CCR5 expression on immune cells and its association with prognosis in melanoma. Given the role of CCR5 in T cell costimulation (112), it would be interesting to understand the relationship between CCR5 expression on T cells and patient outcomes.

### CCR6–CCL20 axis

In melanoma patients, CCR6 was found to be more highly expressed on circulating plasmacytoid DC (pDC) than on pDC found in healthy volunteer controls (105). CCR6-expressing pDC migrated in response to CCL20 stimulation. The presence of CCR6^+^ pDC have been detected in primary melanoma tumors. This infiltration might be in part due to the presence of high concentrations of CCL20, often detected within these primary tumor lesions (105) and mainly produced by tumor-associated macrophages (38). Interestingly, high CCL20 expression is associated with a shorter disease-free period and overall survival of melanoma patients (38). Moreover, given the negative prognostic value conveyed by tumor-infiltrating pDC in melanoma (113), CCR6 is likely to also be associated with poor patient outcome. However, this needs to be explored further and to validated the prognostic value of CCR6^+^pDC in the melanoma tumor microenvironment. We have found that a low proportion of circulating effector memory CD8^+^CCR6^+^ T cells was associated with a better overall survival in stage IV melanoma (106). Collectively, it seems that both CCL20 and CCR6 immune cell expression in multiple cell types are associated with a poor patient outcome (Table 2).

### CCR9–CCL25 axis

CCR9 is expressed at the membrane of several immune cell subsets and is mostly associated with gut homing with the exception of immature T cells in transit from the bone marrow to the thymus (114). Further CCR9^+^ cell populations include intestinal infiltrating T cells (115), gut pDC (116), and small intestinal IgA producing plasma cells (117). Unfortunately, to date, the role of CCR9 expression on immune cells in melanoma and other cancers is poorly understood. We have investigated the impact of CCR9 expression on the membrane of circulating T cells in stage IV melanoma patients. Interestingly, high CCR9 expression on naïve circulating CD8 T cells is associated with a favorable prognosis (106) (Table 2). In mice, we have found tumor infiltrating T cells that express CCR9 and importantly, blockade of its ligand, CCL25, in a sarcoma model, led to increased tumor growth. This is associated with a reduction of CD4^+^ T cell infiltration. Moreover, in this tumor model, high levels of CCL25 were found in the tumor microenvironment and these levels were much higher than the levels found in the gut (106) providing a possible explanation for the recruitment of these CCR9^+^ T cells to the tumor bed. Further studies are warranted to validate this positive impact of CCR9 expression on T cells in this pathology.

### CCR10–CCL27 axis

CCR10 is one of the chemokine receptors that specifically guide the migration of immune cells to the skin in response to the local production and accumulation of CCL27. In contrast to benign lesions where CCL27 is expressed at low levels, many primary melanoma lesions express substantial amounts of this chemokine (44). CCL27 expression is correlated with T lymphocyte density, but unexpectedly, higher chemokine expression is associated with lower T cell infiltrate (44). This suggests that despite the local accumulation of CCL27, CCR10-expressing T cells are unable to infiltrate CCL27-expressing melanoma lesions and these T cells are therefore restricted to circulate in the periphery. Supporting this hypothesis, in our own work we have shown that in stage IV patients, the accumulation of circulating effector memory CCR10 expressing CD4^+^ T cells was associated with shorter overall survival (106). With the exception of these two studies, little is known about the impact of CCR10 expression on immune cells and prognosis. However, it seems that CCL27 tumor concentration was not associated with T cell accumulation and thus their peripheral increase was associated with a poor prognosis (Table 2).

### CXCR3–CXCL9/CXCL10 axis

High expression of CXCR3, on melanoma infiltrating T cells together with the recruitment of effector memory CD8^+^ T cells has been associated with a better patient outcome (87, 89, 109, 118) (Table 2). Mullins et al. (109) reported that high CXCR3 expression on antigen specific CD8^+^CD45RO^+^ T cells is associated with a favorable prognosis in stage III patients but fail to do so in patients with distant metastases (109). We have found that high CXCR3 expression on circulating effector memory CD4^+^ T cells is associated with an enhancement of stage III-IV patient survival, irrespective of tumor lesion location and patient stages (106). Mikucki et al. (110) have demonstrated the critical requirement of CXCR3 expression on mouse CD8^+^ T cells for cell adhesion to, and migration through, the endothelial barrier to infiltrate tumor lesions (110). Furthermore, CXCR3 is associated with Th1/Tc1 polarization and anti-tumor functions (119, 120). Interestingly, therapy such as peptide vaccination in Montanide Adjuvant led to the upregulation of CXCR3 expression on circulating tumor antigen-specific T cells (121) but Hailemichael et al. have shown that most of these CXCR3^+^ T cells induced by the vaccination are retained to the site of vaccine administration (122). Despite this potential induction of CXCR3 expression, CXCR3^+^ T cells are unlikely to reach melanoma lesions in this context. Furthermore, we have found that in stage III/IV patients, CXCR3 is poorly expressed on T cells compared with expression levels observed in healthy volunteers (106). This last observation suggests that (i) CXCR3 is potentially downregulated due to a negative feedback loop of cell regulation following STAT3 activation or (ii) these CXCR3^+^ T cells, which are underrepresented in the periphery, are actually localized to melanoma lesions. Currently, there is little evidence to support either of these two hypotheses. In favor of CXCR3-regulated expression, Yue et al. (123) found that STAT3 expression and signaling mediated CXCR3 downregulation on CD8^+^ T cells thus inhibiting intratumoral CD8^+^ T cell accumulation and impacting anti-tumor functions (123). At steady-state, CXCR3 is tightly regulated at the surface of T cells and downregulation of its expression with or without ligand binding is finely controlled by a regulatory feedback mechanism to preserve cells from over activation (124) and this may even be exacerbated in a pro-inflammatory context. Moreover, we have previously found an enrichment of CXCR3-expressing CD4^+^ T cells in metastatic lymph nodes compared with circulating T cells (106) perhaps explaining the differences found in the blood between melanoma patients and healthy volunteers. In tumor lesions, CXCR3 expression might be sustained by the presence of pro-inflammatory molecules such as IFNγ that has been shown to sustain *Tbx21* expression and subsequently TBET to positively regulate CXCR3 expression at the surface of T cells (125, 126). Together, these studies highlight that the expression of CXCR3 on the surface of T cells is finely regulated and is essential to melanoma infiltration and tumor control. Furthermore, high tumor expression of CXCR3 ligands together with high expression of CXCR3 on T cells are both associated with a favorable prognosis in melanoma (Table 2). Thus, strategies enhancing CXCR3 ligand production or CXCR3 expression on effector and memory T cells, but not melanoma cells, is highly desirable.

### CXCR4–CXCL12 axis

The CXCR4-CXCL12 axis is required for the development and survival of mice as complete deletion of CXCR4 is embryonically lethal (127, 128). This axis plays an essential role in haematopoiesis and cerebellar development, bone marrow immune cell retention and thymic homing (10, 127, 128). To study the role of CXCR4 expression on non-tumor cells and its association with melanoma progression, D'alterio et al. (64) have used CXCR4 heterozygous mice where they intravenously injected CXCR4 expressing B16 melanoma cells. The partial loss of host-CXCR4 expression reduced lung metastases formation that is accompanied by a decrease of CXCL12 concentration together with Ly6G^+^ cell accumulation in lung tissues (64). Similar results have been found in wild type mice treated with a CXCR4 antagonist, Plerixafor (AMD3100) (64). In stage I-III melanoma patients, high expression of CXCR4 in circulating CD4^+^CD45RA^+^ was associated with prolonged disease free survival (Table 2). Moreover, the presence of CXCR4 expressing CD4^+^CD45RA^+^ T cells correlated with absence of primary tumor ulceration (111).

## Do chemokine receptor expression on immune cells reflect the metastatic dissemination of melanoma?

This question was first raised by Salerno et al. (94). They studied whether the expression of organ-specific chemokine receptors and integrins on the surface of T cells differs according to the metastatic site (94). This included the evaluation of CCR4, CCR5, CCR7, CCR9, CXCR3, CLA, and tissue retention integrins on the surface of CD4^+^ and CD8^+^ T cells by flow cytometry. This group found limited evidence that tissue site-specific chemokine receptor expression was associated with the site of metastatic location with the exception of CCR9, which was found to be preferentially expressed on T cells that infiltrate small intestine metastases. Expectedly, the expression of tissue retention integrins was higher on tumor infiltrating T cells than on circulating T cells suggesting a specific maintenance of a pool of intratumoral effector and memory T cells in melanoma lesions (94). This lack of site-specific expression of chemokine receptors on infiltrating T cells might be due in part by an absence of infiltration of these site-specific chemokine receptor-expressing cells. Thus, these cells may be maintained in the circulation. Salerno et al. (94) found that CCR4, CCR5, and CLA are highly expressed on circulating T cells (94). However, how this expression differs from healthy volunteers and to what extent this peripheral expression correlates with site-specific metastases and dictates patient's prognosis were, at this stage, unknown. With this in mind, we retrospectively evaluated the surface expression of nine chemokine receptors and integrins on circulating and tumor infiltrating T cells collected from stage III-IV patients (106). These included the expression of CCR6, CCR7, CCR9, CCR10, CXCR3, CXCR4, CXCR5, CLA, and CD103. Moreover, we studied the expression of the chemoattractant receptor-homologous molecule expressed on Th2 cells, CRTH2, known for its involvement in Th2 polarization and responses (129, 130). When comparing these expression levels to those found on circulating T cells from healthy volunteers, patients with a lower expression of CXCR3 and CCR6 on effector/memory circulating T cells had preferential metastases to the skin and lymph nodes and a decrease of CCR9, together with CXCR4 and CXCR5 expression on both CD4^+^ and CD8^+^ T cells, which was an indicator of the presence of pulmonary lesions (Table 3). In addition, multi-metastatic patients with a broad dissemination of disease displayed an increase of chemokine receptor/integrin expression on naïve T lymphocytes, specifically CCR10, CD103, and CRTH2 (Table 3). This disseminated localization was also associated with a loss of CXCR3 on effector/memory T cells and a decrease in CXCR4 and CCR9 expression on CD4 effector and terminal effector T cells (Table 3). Collectively, these results indicated that the expression pattern of chemokine receptors/integrins on the surface of circulating T cells potentially mirror the metastatic spreading in melanoma patients (106).

**Table 3:** Chemokine receptors expression at the surface of peripheral immune T cells mirrors the melanoma metastatic dissemination.

**Melanoma Stage**	**Tumor lesion localization**	**Chemokine receptors and integrins involved**
Stage III	Regional cutaneous and lymph node metastases	Decrease of CCR6 and CXCR3 expressions on effector/memory peripheral T cells
Stage IV	Regional cutaneous and lymph node metastases + lung metastases	Reduction of CCR9, CXCR4, and CXCR5 expression on circulating T cells
Stage IV	Multi-disseminated disease with or without lung involvement	Increase expression of CCR10, CD103^*^, and CRTH2 on naïve peripheral T cells—Loss of CXCR3 and CCR6 expression on effector and memory circulating T cells—Decrease of CXCR4 and CCR9 expression on effector and terminal effector blood T cells

*Chemokine receptors expression was retrospectively evaluated on circulating blood T cells collected from 57 stage III–IV melanoma patients (131–133)*.

* *Elevated expression of CD103 on naïve T cells is correlated with the presence of liver metastases*.

Interestingly, CD103 expression on naïve T cells was strongly associated with liver metastases (106) suggesting that this integrin might play a role in binding T cells to this organ. CD103 expression is a feature of tissue resident memory T lymphocytes (134) and many T lymphocytes that reside in the gut (115) or the liver (135) express this integrin. Its ligand, E-cadherin, is naturally expressed on hepatocytes (136), and notably in the interlobular bile duct epithelia (137). Shimizu et al. have demonstrated that CD103-expressing CD4^+^ and CD8^+^ T cells accumulated in the liver and these cells harbored a particular phenotype with a decrease of TCRαβ expression (135). As observed in hepatocellular carcinoma (136, 138), a decrease of E-cadherin expression during epithelial-mesenchymal transition of liver metastasis on the surface of hepatocytes is associated with an increase of its soluble form in the serum (139) potentially favoring the circulation of CD103^+^ T cells and their accumulation in the blood of melanoma patients harboring liver metastases (Table 3).

Further retrospective and prospective investigations are warranted to support the clinical relevance of differences in expression of chemokines and chemokine receptors in melanoma. Their evaluation would likely benefit patients in the early detection of metastases and in targeting specific subsets of T cells to favor their migration to desired organs and to target these metastases. Strategies to modulate their expression and functions are needed in order to ameliorate patient prognosis and therapeutic outcomes.

## Potential for targeting

Chemokines and their receptors have dual roles in melanoma and other cancers. On one hand, they promote immune cell recruitment necessary for tumor control (e.g., CXCL9/10/11 and CXCR3). On the other hand, they are involved in tumor escape and metastases formation by (i) selectively guiding tumor cells toward specific organs, which subsequently form secondary lesions (e.g., CCR7 or CXCR4), (ii) favoring the recruitment of immunosuppressive cells (e.g., CCR5) and, (iii) influencing tumor vasculature associated with tumor dissemination (e.g., CXCL10 and CXCR3) (140, 141). Thus, targeting these molecules is of particular interest in melanoma and other cancers as an approach to limit tumor development and to considerably reduce its metastatic spreading. However, the design of selective drugs will need to specifically target tumor cells, the immune system, or both compartments.

Many small molecule antagonists and therapeutic antibodies have been developed (142) but so far, this has led to only a moderate improvement in various diseases. As a consequence, only 3 targeting agents have been approved to treat patients, or are in phase III clinical trials. These include a blocking CCR4 antibody, Mogamulizumab, approved in Japan to treat refractory adult T-cell leukemia, peripheral T cell lymphoma and cutaneous T cell lymphoma (142), an anti-CCR5 antibody tested in graft-vs.-host disease and human immunodeficiency virus-1 (143) and an anti-CXCR4 antibody evaluated in lymphoma and multiple myeloma (144). Thirty-seven additional compounds are currently being tested targeting CCR1, CCR2, CCR3, CCR4, CCR5, CCR9, CXCR1, CXCR2, CXCR4, and CX3C1 (142, 145, 146). In a small study (147), metastatic colorectal cancer patients with CCR5^+^ liver metastases were treated with a small molecule that antagonizes CCR5, Maraviroc, with encouraging results. Therefore, further evaluation in a larger cohort is warranted to determine the benefits and toxicity of this approach.

In melanoma, CXCR4 inhibition with AMD11070 abrogated tumor cell migration in response to CXCL12 stimulation (148). Similarly, the CXCR4 antagonist, AMD3100, prevents the development of squamous cell carcinomas under chronic UV exposure. Mechanistically, UV radiation induced CXCL12 expression in the skin and this was responsible for attracting CXCR4^+^ mast cells. Thus, blocking the CXCR4-CXCL12 pathway using this antagonist reduced mast cell infiltration into the skin, tumors and draining lymph nodes, and this subsequently prevents immune suppression and tumor development (149). Given the involvement of CXCR4 in tumor cell migration to many different organs, oral administration of CXCR4 inhibitors could be particularly efficient. Moreover, CXCR4 is also involved in the recruitment of suppressive immune cells, such as mast cells in the tumor microenvironment.

CCR9 blockade using an antibody significantly reduced the tumor cell migration in response to CCL25 stimulation (42). Interestingly, a new mouse anti-human CCR9 antibody was developed by Somovilla-Crespo et al. showing promising results in blocking the growth of human CCR9^+^ leukemia cells in NSG mice (150). Similarly, the use of the CCR9 antagonist CCX8037 could also specifically interfere with small intestinal dissemination. However, we have shown that the blockade of CCL25 in a sarcoma model inoculated in immunocompetent mice was detrimental and notably, resulted in increasing the tumor growth (106). Further investigations are required to determine the impact of such drugs on both leukocyte trafficking and tumor cell spreading (151) to avoid unexpected off-target effects.

Neonatal skin exposed to UVB induced an IFNγ gene signature response from melanocytes including CCL8 expression (99). Thus accumulation of CCL8 drives the recruitment of CCR2^+^ macrophages that were shown to promote melanomagenesis. The blockade of IFNγ using a specific antibody or the use of CCR2 deficient mice, which were subjected to UVB exposure, have decreased of macrophages infiltration in the skin and reduced tumor volume (99). Similarly, the overexpression of a dominant negative version of CCL2, a non-functional protein that competes with the native form for binding to CCR2, in melanoma tumor bearing mice specifically reduced tumor associated macrophage infiltration that is associated with a decrease of tumor angiogenesis and tumor growth (98). Interestingly, mice inoculated with B16F10 tumors engineered to express GM-CSF harbored an accumulation of monocytic CCR2^+^ MDSC compared to non-GM-CSF expressing tumors. This accumulation of MDSC in melanoma lesions was associated with a reduction of CD8^+^ T cell infiltration and an increase in tumor burden (152). Although vaccination with irradiated B16 cells producing GM-CSF was shown to favor immune responses to immunotherapies in preclinical melanoma models (153, 154), in this setting, this cytokine seemed to play a negative role in antitumor immune surveillance. CCR2 appears to be an attractive target in melanoma and potentially in other tumor types and a CCR2 antibody, plozalizumab, is currently being tested in phase I clinical trial (NCT02723006) in combination with an immune checkpoint blocker, nivolumab.

CRTH2 associated with Th2 responses would be an attractive target in melanoma as this chemokine expression is increased in patients with a multi-metastatic disease (Table 3). CRTH2 is also expressed on eosinophils, basophils, and some monocytes/macrophages (155), immune subsets which all convey a distinct prognosis in melanoma (84, 156). Initially designed for targeting CRTH2^+^ T cells involved in respiratory diseases (157, 158), CRTH2 antagonists could be indicated in multi-metastatic melanoma patients with high CRTH2 expression.

SX-682 (Syntrix Biosystems, Inc) is a selective and potent CXCR1/2 antagonist. CXCR1/2 is expressed on melanoma cells, MDSC and neutrophils and sustains tumor immunosuppression, tumor growth, angiogenesis and tumor dissemination in response to CXCL1, CXCL2 or CXCL8 (107, 108, 159–164) (Tables 1, 2). In melanoma, MDSC accumulated both in tumor lesions and in periphery, correlating with tumor stage. This feature has been associated with a negative prognostic value (84). Furthermore, this compound has been evaluated in combination to anti-CTLA-4 and anti-PD-1 co-blockade in an elegant mouse model of prostate cancer (165). In this model, the authors demonstrated the crucial role of MDSC in sustaining cancer progression. The combination of immune checkpoint inhibitors and SX-682 resulted in decreased prostate mass, lymph node and lung metastases (165). This inhibitor is currently being evaluated in stage III/IV melanoma patients in combination with an anti-PD1 antibody, Pembrolizumab (NCT03161431). This phase I study aims to evaluate the tolerability and safety profile of SX-682 together with the response rate, tumor response duration, progression free and overall survival of the combination. Interestingly, another CXCR1/2 inhibitor, Ladarixin, was shown to significantly reduce human melanoma cell motility and to induce apoptosis *in vitro*. *In vivo* treatment of melanoma xenografts with Ladarixin reduced tumor growth, polarized intratumoral macrophages to M1 phenotype, and inhibited angiogenesis (166). Inhibition of CXCR1/2 appears to be very promising as it targets both melanoma and immune cells, reducing tumor burden alone or in combination with immune checkpoint blockers.

Modulation of chemokine receptor expression on the surface of chimeric antigen receptor (CAR) T or NK cells prior to infusion is promising as this would enhance their tumor infiltration and potentially improve therapeutic results. CX3CR1 genetically modified T cells transferred into CX3CL1 producing colorectal adenocarcinoma tumor bearing mice displayed enhanced tumor infiltration and anti-tumor responses (167). Moreover, significant reduction in tumor size and complete remission have been observed with CCR2b-GD2-CAR T cells and CXCR4-EGFRvIII-CAR NK cells infused in mice bearing CCL2 producing GD2 neuroblastoma or CXCL12 secreting EGFRvIII glioblastoma cells, respectively (168, 169). Similarly, genetically engineered CCR2 expression on CAR T cells directed to the tumor antigen mesothelin increased tumor cell infiltration and anti-tumor responses against large and established tumors inoculated in severe immunodeficient mice (170). To date, CAR specific cells genetically engineered to express particular chemokine receptor have only been tested in preclinical models. Despite having shown impressive anti-tumor responses against primary tumors, it will be challenging to find a chemokine that is highly, specifically and commonly expressed across different tumor microenvironments, found in multi metastatic patients in order to efficiently eradicate all disseminated lesions.

## Conclusion and perspectives

Chemokines and chemokine receptors are key molecules involved in cell migration, proliferation and survival that are critical in maintaining tissue homeostasis. Melanoma cells overexpress many chemokine receptors that are likely involved in cancer progression and metastasis. Thus, modulation of chemokines and chemokine receptors appears to be an attractive target in cancer therapy. However, targeting them is a double edged sword, as treatments will not only affect immune cell migration to tumor lesions or tumor dissemination but also in the long term, impact immune cell development and polarization (e.g., CXCR4). This may partly explain why there is low number of approved drugs targeting chemokines and their receptors in treating chronic diseases, such as cancer. How can we overcome this? In the era of personalized medicine, designing bispecific antibodies that can specifically target a chemokine receptor and a tumor antigen, which are both expressed on the surface of cancer cells is highly attractive. However, antigen escape due to the emergence of tumor variants, which do not express the targeted antigen, are likely to emerge, rendering the treatment ineffective. Another promising area of research is to combine chemokine receptor blockers with anti-PD-1 or anti-CTLA-4 antibodies to further improve the clinical activity of these antibodies and thus further increase patient survival (171). Together, this would lead to reduced tumor infiltration by immunosuppressive cells as Tregs or MDSCs and subsequently, induce anti-tumor immunity by releasing the immunosuppressive brakes. Another approach would be to use engineered antibodies to target privileged metastatic sites. The therapeutic management of brain metastases in melanoma and other cancers is challenging, as the brain is protected by a highly selective blood-brain barrier impermeable to many cells, in particular, immune cells. In melanoma, a bispecific antibody could be designed to target CCR4 and a nanobody, that selectively binds to human cerebromicrovascular endothelial cells. This attached nanobody is then internalized and able to transmigrate across the endothelial barrier (146). As a proof of principle, a bispecific antibody specific for the metabotropic glutamate receptor 1, expressed in the brain, and also carrying a specific nanobody was able to translocate across the endothelial layer into the brain and regulate physiological functions (172).

Given the association between the accumulation of certain chemokines in tumor lesions and the presence of tertiary lymphoid structures, it would be interesting to reinstate chemokine expression in “cold” tumors to favor the emergence of ectopic-like lymphoid organs that are positively associated with immune cell activation and patient survival. Several strategies are currently being tested, aiming to modulate anti-tumor responses through the induction of tertiary lymphoid structures (173).

Collectively, chemokine and chemokine receptors are essential for guiding immune cells to tumor lesions, however melanoma cells often harness these molecules to disseminate to distant organs. Given their broad expression profile and potential side effects, drugs targeting these molecules must be carefully designed. Novel technologies have now rendered this challenge possible with the development of compounds that specifically affect a desired target (145, 146). Many chemokine receptor antagonists are currently being tested in melanoma and other malignancies, if successful, these treatments will diversify the oncologic armamentarium currently available therefore increasing possible therapeutic combinations and ultimately improving patient outcome.

## Author contributions

NJ wrote the initial draft. CD, GB, and LZ made substantial contributions to discussions of the content. All authors reviewed and/or edited the manuscript prior submission.

### Conflict of interest statement

The authors declare that the research was conducted in the absence of any commercial or financial relationships that could be construed as a potential conflict of interest.
